# Psychometrische Eigenschaften der deutschsprachigen Body Image States Scale (BISS)

**DOI:** 10.1055/a-2873-3428

**Published:** 2026-06-19

**Authors:** Tabea Vogel, Kristine Schönhals, Manuel Waldorf, Silja Vocks

**Affiliations:** 1 Klinische Psychologie und Psychotherapie, Universität Osnabrück, Osnabrück, Germany

**Keywords:** Körperbild, Validierung, State-Fragebogen, Körperunzufriedenheit, Body Image, State-Questionnaire, Validation, Body Dissatisfaction

## Abstract

**Ziel der Studie:**

Angesichts der starken Auswirkungen des Körperbildes auf die psychische Gesundheit sind zuverlässige und valide Messinstrumente von großer Bedeutung. Derzeit existiert keine validierte deutsche Version der Body Image States Scale (BISS), sodass in der vorliegenden Studie die BISS, ein Maß für das momentane (state-like) Körperbild, ins Deutsche übersetzt und psychometrisch evaluiert werden sollte.

**Methodik:**

In einer Online-Studie ( *n* =548 weiblich, *n* =41 männlich, *n* =36 nicht-binär/agender) wurden die Faktorenstruktur, Reliabilität und Validität der deutschsprachigen Versionen der BISS untersucht. Um die State-Sensitivität der BISS experimentell zu prüfen, wurden Teilnehmende zufällig einer Bedingung zugeordnet, in der sie sich entweder ein für das Körperbild positives, ein negatives oder kein Szenario vorstellen sollten.

**Ergebnisse:**

Eine Konfirmatorische Faktorenanalyse deutete auf eine einfaktorielle Struktur der BISS hin. Die deutschsprachige Version wies eine gute interne Konsistenz auf, die State-Sensitivität konnte bestätigt werden, außerdem zeigte die BISS eine gute Konvergente Validität mit Maßen der Körperzufriedenheit und den Symptomen von Essstörungen.

**Diskussion:**

Insgesamt zeigte die BISS gute psychometrische Kennwerte, was ihre Eignung für den Einsatz in Forschung und klinischer Praxis im deutschsprachigen Raum nahelegt. Die Verfügbarkeit einer validierten deutschen BISS ermöglicht eine genauere Erfassung momentaner Schwankungen des Körperbildes in klinischen und Forschungssettings.

## Abstract (en)

**Objective:**

Given the strong impact of body image on mental health, reliable and valid assessment tools are crucial. Currently, there is no validated German version of the Body Image States Scale (BISS), a measure of momentary (state-like) body image. Therefore, the present study aimed to translate the BISS into German and to psychometrically evaluate this German version.

**Method:**

An online study (n=548 female, n=41 male, n=36 non-binary/agender) was conducted to assess the factor structure, reliability, and validity of the German-language version of the BISS. To experimentally examine state sensitivity, participants were randomly assigned to imagine a positive body image-related scenario, a negative body image-related scenario, or no scenario.

**Results:**

A confirmatory factor analysis supported a single-factor structure of the BISS. the German-language version demonstrated good internal consistency, state sensitivity, and convergent validity with measures of body satisfaction and eating disorder symptoms.

**Discussion:**

Overall, the BISS exhibited favorable psychometric properties, suggesting its suitability for use in research and clinical practice within German-speaking contexts. The availability of a validated German BISS facilitates a more accurate assessment of momentary body image fluctuations in clinical and research settings.

## Einleitung

Körperunzufriedenheit wird häufig im Kontext von Essstörungen untersucht und korreliert in hohem Maße mit deren Entstehung und Aufrechterhaltung 1 2 . Darüber hinaus findet sich ein negatives Körperbild oft auch bei Personen ohne Essstörungen 3 4 5 6 und steht z. B. im Zusammenhang mit Depression 3 , Angst 3 , Körperdysmorpher Störung 2 , einer reduzierten Lebensqualität 4 , einem reduzierten Selbstwert 1 3 und einem erhöhtem Body Mass Index (BMI) 5 . Es wird zwischen dem überdauernden (trait-like) und dem momentanen (state-like) Körperbild differenziert 7 . Personen, die ein negativeres überdauerndes Körperbild haben, tendieren dazu, auch im momentanen Körperbild negativere Werte aufzuweisen 8 . Menschen mit einem negativeren überdauernden Körperbild zeigen jedoch keine stärker ausgeprägten Schwankungen ihres momentanen Körperbildes als Personen mit einem positiveren überdauernden Körperbild. Sowohl das überdauernde als auch das momentane Körperbild sind ein Prädiktor für Essstörungspathologie 8 . Während hauptsächlich Instrumente zur Erfassung des trait-like Körperbilds verfügbar sind, besteht ein Mangel an validierten Fragebögen zur Erfassung des state-like Körperbilds 9 . Im englischsprachigen Raum wurde die Body Image States Scale (BISS) 7 entwickelt, um diese Lücke zu schließen. Die BISS erfasst das state-like Körperbild, ist gut etabliert und wurde bereits in verschiedene Sprachen übersetzt 10 11 . Bislang wurden in europäischen Validierungen jedoch keine Untersuchungen zur Sensitivität der BISS für kurzfristige Veränderungen des Körperbildes durchgeführt 10 11 , weshalb unklar bleibt, ob diese Übersetzungen momentane Veränderungen der Körperzufriedenheit tatsächlich erfassen können. Um kurzfristige Veränderungen der Körperzufriedenheit durch alltägliche Auslöser, wie beispielsweise den Konsum von Social Media oder auch um die direkten Effekte von psychotherapeutischen Interventionen wie z. B. Körperkonfrontationen abzubilden, werden Maße wie die BISS auch im deutschsprachigen Raum benötigt 12 13 14 15 . Die Messung von Veränderungen in überdauernden Merkmalen (z. B. trait-like Körperunzufriedenheit) hilft zwar, beispielsweise die Wirksamkeit einer Therapieform im Vergleich zu einer anderen zu überprüfen, momentane Merkmalsveränderungen könnten jedoch relevante Hinweise auf Veränderungsmechanismen geben, die bestimmten Behandlungen möglicherweise zu Grunde liegen 16 . Entsprechend ist es für die Psychotherapieforschung relevant, ein deutschsprachiges Messinstrument zur Erfassung des momentanen Körperbildes bereit zu stellen und hinsichtlich seiner Gütekriterien zu überprüfen.

Die BISS besteht aus 6 Items, welche auf einer 9-stufigen Likert-Skala bewertet werden und bildet das Spektrum von Körperunzufriedenheit bis Körperzufriedenheit ab. Höhere Scores der BISS deuten auf eine höhere Körperzufriedenheit hin. Die gute interne Konsistenz (0,77≤α≤0,92) sowie die einfaktorielle Struktur der BISS konnte bereits in zahlreichen Kontexten nachgewiesen werden 7 10 11 . Auch die konvergente und differentielle Validität konnten in der Originalstudie sowie in französisch- und spanischsprachigen Validierungsstudien bestätigt werden 7 10 11 .

Ziel der vorliegenden Studie war die psychometrische Überprüfung der deutschsprachigen Fassung der BISS, mit einem besonderen Fokus auf der Untersuchung der State-Sensitivität mittels einer Imaginationsaufgabe, orientiert an der Originalstudie, da die State-Sensitivität 7 in anderen Validierungsstudien zur BISS bislang vernachlässigt wurde 10 11 . Es wurde erwartet, dass der Score der BISS negativ mit Maßen für trait-like Körperunzufriedenheit, Essstörungssymptomatik und körperdysmorphen Symptomen korreliert und positiv mit dem state-like Selbstwert bezogen auf das Aussehen.

## Methodik

### Stichprobe und Rekrutierung

Es wurde eine Online-Erhebung in 2 Teilen durchgeführt. Teil 1 wurde von *N=* 628 Teilnehmenden vollständig bearbeitet ( *n* =548 weiblich, *n* =41 männlich und *n* =36 non-binär/agender). Davon nahmen *n* =402 Personen ( *n* =356 weiblich, *n* =26 männlich, *n* =19 non-binär/agender) ca. 2 Wochen später an Teil 2, der Retest-Erhebung der BISS, teil. Das Alter der Teilnehmenden lag zwischen 16 und 72 Jahren ( *M* =27,51, *SD* =10,17). Der durchschnittliche BMI betrug *M* =24,23 kg/m² ( *SD* =6,19). 51% der Teilnehmenden gaben ein Abitur als höchsten Bildungsabschluss an. Weitere Angaben zu den Häufigkeiten der Bildungsabschlüsse für die Teilnehmenden von Teil 1 und Teil 2 der Studie sind dem Zusatzmaterial 1 zu entnehmen. Zu beachten ist, dass die Retest-Erhebung an derselben Stichprobe durchgeführt wurde wie Teil 1 der Studie, wobei jedoch nicht alle Teilnehmenden von Teil 1 auch an Teil 2 teilnahmen. Personen, die an beiden Teilen der Studie teilnahmen, konnten sich für eine Gutscheinverlosung anmelden. Die Rekrutierung erfolgte über Flyer und E-Mail-Verteiler an der Universität Osnabrück sowie durch Werbung auf Instagram.

### Messinstrumente

#### Body Image States Scale (BISS; 7 )

Die BISS besteht aus 6 Items, welche auf einer Skala von 1 (z. B. „äußerst unzufrieden“) bis 9 (z. B. „äußerst zufrieden“) eingeschätzt werden. Höhere BISS Werte indizieren hierbei eine höhere Körperzufriedenheit. Die BISS wurde in Anlehnung an eine im deutschsprachigen Raum verwendete, jedoch noch nicht validierte Version 13 17 ins Deutsche übersetzt und durch eine muttersprachlich englische Fachübersetzerin rückübersetzt. Diskrepanzen in der Übersetzung wurden in einem Expert*innenteam diskutiert und konsensbasiert gelöst. In Übereinstimmung mit den Empfehlungen einer französischen Validierungsstudie 10 und der Entscheidung des Expert*innenteams wurde Item 2 („body size and shape“) mit „Figur“ übersetzt (siehe Item 5, Zusatzmaterial 2). Die deutschsprachige Übersetzung wurde in Anlehnung an andere Validierungsstudien (F-BISS und S-BISS) „G-BISS“ („German-BISS“) genannt 10 11 . Die Reihenfolge der Items wurde dahingehend geändert, dass die körpergewichtsabhängigen Items zuletzt gezeigt wurden, um bei den Berechnungen zu einer ursprünglich geplanten zusätzlichen körpergewichtsunabhängigen 4-Item-Version auszuschließen, dass die körpergewichtsabhängigen Items die Reaktionen auf die darauffolgend dargebotenen Items beeinflussen könnten. Zudem wurde das veränderte Frage-Antwort-Format der BISS aus der im deutschsprachigen Raum gängigen Übersetzung der BISS 17 übernommen. Da die BISS ein sehr kurzes State-Instrument ist und invertierte Items zu vermehrten Antwortfehlern führen könnten 18 , wurde durch einen Expert*innen-Konsens entschieden, die Invertierung einiger der ursprünglichen Items in der deutschsprachigen Version nicht beizubehalten. Stattdessen wurden alle Items in nicht invertierter Form dargeboten (siehe Zusatzmaterial 2).

#### Eating Disorder Examination Questionnaire (EDE-Q; 19 )

Der EDE-Q wurde zur Erfassung der spezifischen Pathologie von Essstörungen eingesetzt. Der Gesamtfragebogen mit 22 Items weist in dieser Studie eine gute interne Konsistenz auf ( *α* =0,96, *ω* =0,96). Höhere Werte indizieren eine stärker ausgeprägte Essstörungssymptomatik. Auch die konvergente Validität des EDE-Q konnte bestätigt werden 19 .

#### Eating Disorder Inventory-2 (EDI-2; 20 )

Die Subskala *Körperunzufriedenheit* des EDI-2 wurde zur Erfassung des Konstrukts der allgemeinen Unzufriedenheit mit dem Körper verwendet. Diese umfasst 9 Items, welche mittels einer 6-stufigen Likert-Skala eingeschätzt werden. Sie weist in dieser Studie eine gute interne Konsistenz ( *α=* 0,91, *ω* =0,91) auf. Höhere Werte indizieren eine stärker ausgeprägte Körperunzufriedenheit. Die konvergente Validität wurde belegt 21 .

#### Fragebogen Körperdysmorpher Symptome (FKS; 22 )

Der FKS dient der Erfassung körperdysmorpher Symptome, welche auf das Vorliegen einer Körperdysmorphen Störung (KDS) hindeuten können. Verwendet wurde die Subskala Spezifische KDS-Symptome. Sie umfasst 15 Items, welche mittels einer 5-stufigen Likert-Skala eingeschätzt werden. Höhere Werte indizieren hierbei eine stärker ausgeprägte Symptomatik. Die Subskala weist in dieser Studie eine gute interne Konsistenz ( *α* =0,91, *ω* =0,92) auf. Die diskriminante Validität des Fragebogens wurde bestätigt (KDS / nicht KDS) 22 .

#### Soziodemografischer Fragebogen

Für die Charakterisierung der Stichprobe wurden das Alter, die Geschlechtsidentität, der höchste Bildungsabschluss, die Größe, das Gewicht sowie das Vorliegen einer Essstörungsdiagnose (Selbstbericht) erfasst.

#### State Self-Esteem Scale (SSES; 23 )

Die SSES misst kurzzeitige Veränderungen des Selbstwertgefühls. Für die vorliegende Studie wurde zur Erfassung des momentanen Selbstwertgefühls bezüglich des Aussehens die entsprechende Subskala des Fragebogens, bestehend aus 5 Items, genutzt. Sie zeigte in dieser Studie eine gute interne Konsistenz ( *α* =0,87, *ω* =0,88). Die konvergente Validität des Fragebogens konnte bestätigt werden 23 . Niedrigere Werte indizieren eine niedrigere Selbsteinschätzung des Aussehens.

#### Imaginationsexperiment (State-Sensitivität)

Die State-Sensitivität der BISS wurde analog zur Originalstudie 7 überprüft. Die Teilnehmenden wurden in eine von 3 Bedingungen randomisiert. In der Bedingung „positive Imagination“ bzw. „negative Imagination“ wurde ihnen entweder eine hinsichtlich der erwarteten Effekte auf das Körperbild positive oder negative Imaginationsaufgabe angezeigt. Die Teilnehmenden sollten sich vorstellen wie sie sich in der entsprechenden Situation fühlen würden, woraufhin sie dann die G-BISS erneut ausfüllten. In der positiven Bedingung sollten sich die Teilnehmenden vorstellen, dass sie auf einer Party sind und ihnen Komplimente für ihr Aussehen gemacht werden. In der negativen Bedingung hingegen wurden die Teilnehmenden instruiert sich vorzustellen, dass sie alleine in einer Social-Media-App scrollen und schlanke und durchtrainierte Models sehen. In der neutralen Kontrollbedingung wurde keine Imaginationsaufgabe eingesetzt. Hier wurden Teilnehmende lediglich instruiert, die G-BISS erneut auszufüllen. Für eine detaillierte Beschreibung des Vorgehens siehe Zusatzmaterial 3. Die negative Vorstellungsaufgabe wurde im Vergleich zur Originalstudie 7 leicht modifiziert, um eine höhere Aktualität zu gewährleisten. Anstatt sich vorzustellen, in einem Magazin zu lesen, wurde imaginativ in einer Social-Media-App gescrollt.

### Durchführung

Die Teilnehmenden gelangten über einen Link oder einen QR-Code zu einer LimeSurvey-Umfrage (Version: 6.4.2+240115). Dort wurden sie zunächst über den Studieninhalt und -ablauf aufgeklärt und gaben ihr Einverständnis zur Teilnahme. Anschließend wurden soziodemographische Fragen beantwortet und die G-BISS sowie der SSES ausgefüllt. Im Anschluss wurden Teilnehmende in eine der 3 Bedingungen randomisiert. Sie wurden entweder zu einer für das Körperbild positiven oder negativen Vorstellungsaufgabe angeleitet oder ihnen wurde in der neutralen Bedingung keine Vorstellungsaufgabe gezeigt. Danach füllten Sie erneut die G-BISS aus. Im Anschluss beantworteten sie den FKS, den EDI-2 und den EDE-Q. Teilnehmende, die an der Retest-Erhebung zwei Wochen später, interessiert waren, konnten in einer getrennten Umfrage ihre E-Mail-Adresse angeben. Die Retest-Erhebung bestand aus dem erneuten Ausfüllen der G-BISS. Die Durchführung der Studie wurde von der Ethikkommission der Universität Osnabrück genehmigt (Az.: 4/71043.5).

### Statistische Analysen

Alle Berechnungen, mit Ausnahme der Konfirmatorischen Faktorenanalyse wurden in IBM SPSS (Version 28) durchgeführt. Die Konfirmatorische Faktorenanalyse wurde in RStudio (Version 4.4.1, lavaan-Paket) mittels Strukturgleichungsmodellierung mit robuster Maximum-Likelihood-Schätzung mit Satorra-Bentler Korrektur (SB) durchgeführt, da der Kolmogorov-Smirnov Test auf eine Verletzung der Normalverteilungsannahme bei allen Items hinwies (alle p<0,001). Als Grenzwerte für einen akzeptablen Modellfit wurden CFI≥0,90, TFI≥0,90, RMSEA≤0,08 und SRMR≤0,10 sowie für einen guten Modellfit CFI≥0,95, TFI≥0,95, RMSEA≤0,06 und SRMR≤0,08 herangezogen 24 . Explorativ wurden Modifikationsindizes berechnet, um mögliche Respezifikationen für das Modell vorzuschlagen.

Die Konvergente Validität der G-BISS wurde durch Produkt-Moment-Korrelationen zwischen dem Score der G-BISS und den Scores von EDE-Q, EDI-2, SSES und FKS überprüft. Außerdem wurde mittels eines ungepaarten *t* -Tests geprüft, ob es einen signifikanten Unterschied in den G-BISS-Scores von Personen mit und ohne Essstörung gab. Wenn keine Varianzhomogenität gegeben war, wurde ein Welch-Test verwendet. Außerdem wurde Cohens *d* berechnet. Effektstärken um *|d|* =0,2 wurden als kleine, um | *d* |=0,5 als mittlere und um | *d* |=0,8 als große Effekte eingeordnet 25 . Die interne Konsistenz wurde mittels Cronbachs α und McDonalds *ω* berechnet. Ein Cronbachs *α* wurde bei *α* >0,90 als exzellent, bei *α* >0,70 als akzeptabel und bei *α* >0,50 als inakzeptabel interpretiert 26 , während für McDonalds *ω* Werte von *ω* >0,65 – 0,80 als akzeptabel und *ω>* 0,80 als exzellent eingeordnet wurden 27 . Für das partielle Eta-Quadrat wurde für um η *²*_*p*_ =0,01 als kleiner Effekt, η *²*_*p*_ =0,06 als mittlerer und η *²*_*p*_ =0,14 als großer Effekt eingeordnet 25 . Für die Retest-Reliabilität wurden Produkt-Moment Korrelationen der G-BISS-Scores zu Zeitpunkt 1 vor der Imaginationsaufgabe und Zeitpunkt 2 (zwei Wochen später) berechnet. Die State-Sensitivität der G-BISS wurde durch eine zweifaktorielle ANOVA mit Messwiederholung und 3 gepaarten *t* -Tests überprüft. Diese wurden jeweils mit Bonferroni korrigiert. Als Alphafehlerniveau wurde *α=* 0,05 zugrunde gelegt.

## Ergebnisse

### Faktorstruktur

**Abb. 1:**
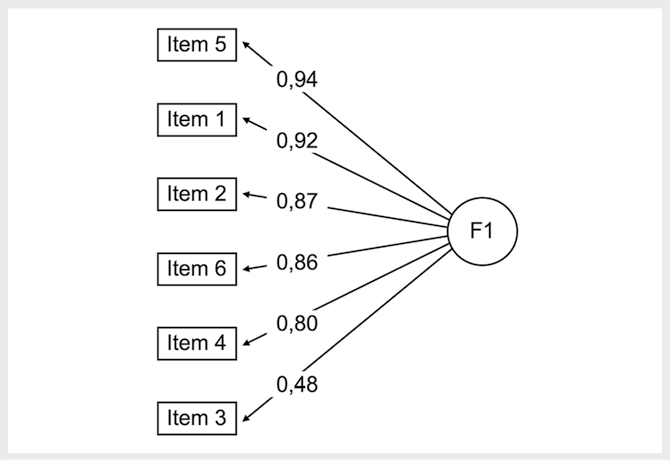
Ladungen der G-BISS Items in der konfirmatorischen Faktorenanalyse. Standardisierte Ladungen auf Basis einer Strukturgleichungsmodellierung mit robuster Maximum-Likelihood-Schätzung mit Satorra-Bentler Korrektur. Abkürzungen: F1, Faktor 1.

Die Konfirmatorische Faktorenanalyse wies eine signifikante Chi-Quadrat-Teststatistik *χ²* (9)=152,41, *p* <0,001 auf. Der CFI=0,944 und TLI=0,906 deuteten auf einen akzeptablen Modellfit, der RMSEA=0,181 (90% KI [0,157, 0,207]) auf einen schlechten Modellfit und der SRMR=0,035 auf einen guten Modellfit hin. Die Ladungen der Items sind Abb. 1 zu entnehmen. Die Itemkennwerte (Mittelwerte, Standardabweichungen, Schiefe und Kurtosis) sind in Zusatzmaterial 4 zu aufgeführt. Die explorativ analysierten Modifikationsindizes (MIs) wiesen auf eine substantielle Verbesserung des Modellfits hin, wenn die Fehlervarianzen der Items 5 „Genau in diesem Moment bin ich mit meiner Figur…“ und 6 „Genau in diesem Moment bin ich mit meinem Körpergewicht…“ miteinander korrelieren dürfen, wobei der RMSEA=0,114 dennoch weiterhin auf einen schlechten Modellfit hindeutet. Weitere mögliche Respezifikationen auf Grundlage der MIs weisen auf eine deutliche Verbesserung des Modellfits hin, wenn zusätzlich die Fehlervarianzen der Items 2 „Genau in diesem Moment fühle ich mich körperlich…“ und 4 „Genau in diesem Moment fühle ich mich hinsichtlich meines Aussehens im Vergleich zu einer durchschnittlichen Person…“ sowie der Items 4 und 5 korrelieren dürfen, wobei der RMSEA = 0,070 dann auf eine akzeptable Modellgüte hindeutet. Eine Übersichtstabelle ist Zusatzmaterial 5 zu entnehmen.

### Reliabilität

Es zeigte sich eine gute interne Konsistenz mit einem exzellenten *α* =0,92, und einem exzellenten *ω* =0,94 sowie eine gute Retest-Reliabilität von *r* =0,81, *p* =0,001.

### Validität

#### Konvergente Validität

Die G-BISS Scores korrelierten signifikant (alle *p* <0,01) in die erwartete Richtung mit der Subskala *Selbstwertschätzung Aussehen* der SSES, der Subskala *Körperunzufriedenheit* des EDI-2, dem Gesamtscore des EDE-Q und der Subskala *Spezifische KDS-Symptomatik* des FKS (siehe Tab. 1 ).

**Table:** **Tab. 1** Korrelationen von G-BISS und konvergenten Maßen

**Konvergentes Maß**	**G-BISS**
**EDI-2**	–0,790**
**EDE-Q**	–0,741**
**SSES**	0,833**
**FKS**	–0,673**

Berechnet wurden Produkt-Moment-Korrelationen. Abkürzungen: G-BISS, Body Image States Scale German; EDE-Q, Eating Disorder Examination-Questionnaire; EDI-2, Eating Disorder Inventory-2; FKS, Fragebogen Körperdysmorpher Symptome; SSES, State Self-Esteem Scale. **p<0,01.

#### Differenzielle Validität

Teilnehmende, die angaben, an einer Essstörung erkrankt zu sein ( *n=* 82, *M=* 2,62, *SD* =1,21, 89% weiblich, 11% non-binär/agender) erzielten signifikant *t* (134,8)=–16,9, *p* <0,001 (95% KI [–2,86, –2,26]) niedrigere G-BISS Scores als Teilnehmende, die dies verneinten ( *n* =546, *M=* 5,18, *SD* =1,67). Es ergab sich eine große Effektstärke (Cohens *d* =–1,5).

Es zeigte sich kein signifikanter Zusammenhang zwischen dem Alter der Teilnehmenden und den BISS-Scores ( *r* =–0,066, *p* =0,10).

### State-Sensitivität

Für den Vergleich der Prä-Scores der G-BISS vor der positiven bzw. negativen Imaginationsaufgabe oder neutralen Kontrollbedingung ohne Imaginationsaufgabe mit den Post-Scores der G-BISS nach der jeweiligen Aufgabe ergab sich eine signifikante Interaktion zwischen den Faktoren Bedingung und Zeitpunkt *F* (2, 625)=261,34, *p* <0,001, mit einem mittleren Effekt *η²*_*p*_ =0,46 25 . Drei gepaarte post-hoc *t* -Tests ergaben, dass die BISS-Scores bei der Post-Messung in der positiven Bedingung signifikant höher waren als bei der Prä-Messung *t* (225)=16,30, *p* <0,001, mit einer großen Effektstärke von *d* =1,08. In der neutralen Bedingung hingegen ergab sich kein signifikanter Unterschied von der Prä- zur Postmessung, *t* (192)=–1,64, *p* =0,309, mit einer sehr kleinen Effektstärke von *d=* –0,12. In der negativen Bedingung nahmen die Scores der G-BISS signifikant ab, *t* (208)=–10,78, *p* <0,001, mit einer großen Effektstärke von *d* =–0,75 (Siehe Tab. 2 für Mittelwerte und Standardabweichungen).

**Table:** **Tab. 2** Deskriptive Statistiken zu den G-BISS Scores prä und post Imaginationsaufgabe

	Prä BISS-Score		Post BISS-Score		
Bedingung	*M*	*SD*	*M*	*SD*	*d*
**Positiv**	5,06	1,86	6,45	1,76	1,08
**Neutral**	4,56	1,83	4,51	1,82	–0,12
**Negativ**	4,87	1,85	4,04	1,83	–0,75

Abkürzungen: G *-* BISS, German Body Image States Scale; *M* , Mittelwert; *SD* , Standardabweichung; *d* , Cohens *d.*

## Diskussion

Ziel der vorliegenden Studie war die psychometrische Überprüfung einer deutschsprachigen Version der BISS. Insgesamt deuten die Ergebnisse auf gute psychometrische Kennwerte der G-BISS hin. Wie in anderssprachigen Validierungsstudien 10 11 deuten die Faktorenanalysen auf eine einfaktorielle Struktur der G-BISS hin. Es wurde jedoch deutlich, dass sich Item 3 auf Grund seiner niedrigen Ladung von den anderen Items zu unterscheiden scheint. Diese Abweichung ist möglicherweise darauf zurückzuführen, dass sich die Itemanker von denen der anderen Items unterschieden (Item 3: „sehr viel schlechter/besser als gewöhnlich“, Beispiel anderer Items: „äußerst unzufrieden/zufrieden“). Item 3 ist das einzige Item, in dem die Teilnehmenden eine Angabe dazu machen sollen, wie sie sich im Moment im Vergleich zu üblicherweise fühlen. Dies erklärt möglicherweise die niedrige Ladung in der Konfirmatorischen Faktorenanalyse.

Die Retest-Reliabilität in der vorliegenden Studie ( *r* =0,81) ist deskriptiv etwas höher als in der englischsprachigen Originalstudie 7 ( *r* =0,69), befindet sich jedoch numerisch nah an den Ergebnissen ( *r* =0,86) aus einer französischen Validierungsstudie 10 . Diese Unterschiede zur Originalstudie 7 sind möglicherweise auch auf die leicht veränderte Durchführung zurückzuführen, so füllten Teilnehmende den Fragebogen beispielsweise online und nicht im Labor aus 10 .

Im Hinblick auf die Konvergente Validität konnte festgestellt werden, dass die G-BISS negativ mit den Scores der Subskala *Körperunzufriedenheit* des EDI-2 korrelierte. Auch mit dem Gesamtscore des EDE-Q korrelierte die G-BISS negativ. Diese Ergebnisse ( *r* =0,74) stimmten mit denen aus einer französischen Validierungsstudie ( *r* =0,72) der BISS 10 annähernd überein. Mit den Scores der Subskala *Selbstwertschätzung des Aussehens* des SSES korrelierten die G-BISS Scores erwartungsgemäß positiv. Die Scores der Subskala *Spezifische KDS-Symptome* des FKS korrelierten negativ mit den BISS Scores. Diese Ergebnisse deuten darauf hin, dass die G-BISS Körperunzufriedenheit valide erfassen kann. Ähnlich wie bei einer spanischen BISS-Validierung 11 zeigte sich ein signifikanter Unterschied in den BISS-Scores von Teilnehmenden, die angaben, aktuell an einer Essstörung erkrankt zu sein, im Vergleich zu denen, die dies verneinten. Auch die Untersuchung der internen Konsistenz der G-BISS ergab mit anderen Validierungsstudien ebenfalls gut vergleichbare Ergebnisse (vorliegende Studie: *α* =0,92; Originalstudie: *α* =0,77; französische Validierung: *α* =0,83; spanische Validierung: *α* =0,92) 7 10 11 . Dies deutet darauf hin, dass die G-BISS zwischen Personen mit und ohne Essstörung die zu erwartenden Unterschiede anzeigt. Aufgrund der geringen Anzahl von Teilnehmenden, die sich als männlich oder non-binär/agender identifizieren, konnten mögliche Unterschiede zwischen den Geschlechtsidentitäten nicht untersucht werden. Entsprechend sind die Ergebnisse dieser Studie im Hinblick auf sich als männlich oder non-binar/agender identifizierende Menschen zurückhaltend zu interpretieren. Berechnungen der Analysen nur mit sich als weiblich identifizierenden Teilnehmerinnen zeigten nur minimale Unterschiede im Nachkommastellenbereich im Vergleich zu den in dieser Studie genutzten Ergebnisse für die Gesamtstichprobe, sodass letztere berichtet wurden.

Die experimentelle Überprüfung der State-Sensitivität konnte zeigen, dass eine für das Körperbild positive Imaginationsaufgabe mit einem signifikant höheren G-BISS-Score einherging. Erwartungsgemäß ging eine negative Imaginationsaufgabe mit einem signifikant niedrigeren G-BISS-Score einher. Dieses Ergebnis stimmt mit den Erkenntnissen der Originalstudie 7 überein. Beachtet werden sollte jedoch, dass das Vorgehen in der vorliegenden Studie von dem Vorgehen der Originalstudie dahingehend abwich, dass es nur eine positive, eine neutrale und eine negative Bedingung gab. In der Originalstudie 7 waren 2 positive und 2 negative Imaginationsaufgaben eingesetzt worden. Zudem wurde die Vorstellungsaufgabe der negativen Bedingung leicht modernisiert, sodass statt „in einer Zeitschrift geblättert“, „in einer Social-Media-App gescrollt“ wurde.

Bei der Interpretation der Daten sind einige Limitationen zu berücksichtigen. Die Reihenfolge der G-BISS Items wurde geändert, um bei den Rechnungen zu einer ursprünglich geplanten körpergewichtsunabhängigen Version auszuschließen, dass die körpergewichtsabhängigen Items (im Original Item 2 und 3) einen Einfluss auf Reaktionen auf die darauffolgend dargebotenen Items haben. Zudem wurde das veränderte Frage-Antwort-Format der BISS aus der im deutschsprachigen Raum gängigen Übersetzung der BISS 17 übernommen. Außerdem wurde entschieden, die Invertierung einiger der ursprünglichen Items in der deutschsprachigen Version nicht beizubehalten. Es ist nicht auszuschließen, dass diese Veränderungen einen Einfluss auf die Ergebnisse dieser Studie hatten. Zudem konnten nicht genügend Personen, welche sich als männlich oder non-binär/agender identifizierten, rekrutiert werden, um beispielsweise die State-Sensitivität der G-BISS für verschiedene Geschlechtsidentitäten zu untersuchen. Um die Zuverlässigkeit der G-BISS für sich nicht als weiblich identifizierende Personen zu belegen, sollte zukünftig eine erneute Validierung mit sich als männlich oder non-binär/agender identifizierenden Personen durchgeführt werden. Auch die „Modernisierung“ der negativen Vorstellungsaufgabe stellt eine Veränderung der Studiendurchführung im Vergleich zum Original dar und erschwert den Vergleich mit anderen Validierungsstudien der BISS. Auch sollte beachtet werden, dass die explorativen Respezifikationen zeigen, dass die Modell-Fit-Indizes deutlich verbessert werden können, wenn Fehlervarianzen ausgewählter Items korreliert werden. Da diese Modifikation jedoch auf den Modifikationsindizes der vorliegenden Stichprobe basieren, liegt nur eine eingeschränkte Aussagekraft vor. Es wäre sinnvoll, in Zukunft zusätzlich eine Überprüfung mit einer unabhängigen Stichprobe durchzuführen.

Trotz dieser Limitationen weist die Studie Stärken auf. So könnte beispielsweise die „Modernisierung“ der Vorstellungsaufgaben dazu beigetragen haben, dass ein Bezug zur Lebensrealität der Teilnehmenden hergestellt wurde. Zudem konnte nachgewiesen werden, dass die G-BISS kurzfristige Veränderungen der Körperzufriedenheit erfassen kann.

## Fazit für die Praxis

Die G-BISS stellt ein valides und reliables Instrument zur Erfassung kurzzeitiger, situational bedingter Veränderungen der Körperzufriedenheit im deutschsprachigen Raum dar. Auch die klinisch-psychologische Praxis kann durch die G-BISS profitieren, indem Veränderungen der Körperzufriedenheit durch Interventionen im Rahmen von Therapien erfasst werden können. Die G-BISS weist gute psychometrischen Kennwerte auf und ist ab 16 Jahren für den deutschsprachigen Raum empfehlenswert.

## Erklärung zur Datenverfügbarkeit

Die für diese Studie erstellten Datensätze sind auf begründete Anfrage bei der Erstautorin erhältlich.

## Beiträge der Autorinnen

TV und SV: Planung der Studie. TV: Technische Durchführung und Datenerhebung. TV und KS: Datenanalyse. TV, KS, MW und SV: Erstellung des Manuskripts. Alle Autor*innen haben an der Erstellung des endgültigen Manuskripts mitgewirkt und die eingereichte Version genehmigt.

## Zusätzliches Material

Zusätzliches Material Zusätzliches Material
