# Supplementary material for: Psychometric Properties of the German Body Image States Scale (BISS)
Source: Psychother Psychosom Med Psychol. 2026 Jun 19;76(8):348–54. [Article in German] doi: 10.1055/a-2873-3428 (PMC13481115; doi:10.1055/a-2873-3428)
Supplement: Supplementary file 1 [file 10-1055-a-2873-3428-1588-02-2026-dv.pdf]

Zusatzmaterial

Zusatzmaterial 1
Bildungsabschlüsse der Teilnehmenden

Schulabschluss	Gesamtstichprobe (%)	Re-Test Stichprobe (%)
Hauptschulabschluss	0,2	0
Realschulabschluss (Mittlere Reife)	7,7	6
Fachhochschulreife	6,2	6
Abitur (Allgemeine Hochschulreife)	51	54
Fachhochschulabschluss	8,3	7,3
Universitätsabschluss	26,1	26,5
Keine Angabe	0,5	0,2

Zusatzmaterial 2
G-BISS

Bitte kreuzen Sie für jede der unten genannten Aussagen die Antwort an, welche am besten beschreibt, wie Sie sich **jetzt, genau in diesem Moment** fühlen. Lesen Sie sich die Aussagen dazu sorgfältig durch.

1. **Genau in diesem Moment bin ich mit meiner körperlichen Erscheinung …**
    - äußerst unzufrieden.
    - größtenteils unzufrieden.
    - mittelmäßig unzufrieden.
    - etwas unzufrieden.
    - weder zufrieden noch unzufrieden.
    - etwas zufrieden.
    - mittelmäßig zufrieden.
    - größtenteils zufrieden.
    - äußerst zufrieden.

2. **Genau in diesem Moment fühle ich mich körperlich …**
    - äußerst unattraktiv.
    - sehr unattraktiv.
    - mittelmäßig unattraktiv.
    - etwas unattraktiv.
    - weder attraktiv noch unattraktiv.
    - etwas attraktiv.
    - mittelmäßig attraktiv.
    - sehr attraktiv.
    - äußerst attraktiv.

3. **Genau in diesem Moment fühle ich mich hinsichtlich meines Aussehens …**
    - sehr viel schlechter als gewöhnlich.
    - viel schlechter als gewöhnlich.
    - etwas schlechter als gewöhnlich.
    - nur wenig schlechter als gewöhnlich.
    - ungefähr so wie immer.
    - nur wenig besser als gewöhnlich.
    - etwas besser als gewöhnlich.
    - viel besser als gewöhnlich.
    - sehr viel besser als gewöhnlich.

4. **Genau in diesem Moment fühle ich mich hinsichtlich meines Aussehens im Vergleich zu einer durchschnittlichen Person …**
   - sehr viel schlechter.
   - viel schlechter.
   - etwas schlechter.
   - nur wenig schlechter.
   - weder besser noch schlechter.
   - nur wenig besser.
   - etwas besser.
   - viel besser.
   - sehr viel besser.

5. **Genau in diesem Moment bin ich mit meiner Figur …**
   - äußerst unzufrieden.
   - größtenteils unzufrieden.
   - mittelmäßig unzufrieden.
   - etwas unzufrieden.
   - weder zufrieden noch unzufrieden.
   - etwas zufrieden.
   - mittelmäßig zufrieden.
   - größtenteils zufrieden.
   - äußerst zufrieden.

6. **Genau in diesem Moment bin ich mit meinem Körpergewicht …**
   - äußerst unzufrieden.
   - größtenteils unzufrieden.
   - mittelmäßig unzufrieden.
   - etwas unzufrieden.
   - weder zufrieden noch unzufrieden.
   - etwas zufrieden.
   - mittelmäßig zufrieden.
   - größtenteils zufrieden.
   - äußerst zufrieden.

Zusatzmaterial 3

Vorgehen Imaginationsaufgaben

Die Imaginationsaufgaben wurden folgendermaßen eingeleitet: „Stellen Sie sich bitte die folgende Situation vor und füllen Sie den nachfolgenden Fragebogen so aus, wie Sie denken, dass Sie sich in der beschriebenen Situation fühlen würden. Versuchen Sie sich die Situation ganz genau vorzustellen, wie fühlt sich Ihr Körper an?“

Positive Bedingung: „Sie sind auf einer Party mit einigen Freunden und Bekannten. Im Laufe der Party bemerken einige Ihrer Freunde Ihr Aussehen. Sie machen Ihnen Komplimente und sagen Ihnen wie gut Sie in dem was Sie an dem Abend tragen aussehen.“

Negative Bedingung: „Sie sind allein in Ihrem Schlafzimmer und scrollen durch eine Social Media App. Sie sehen Inhalte zu Mode und Fitness. Sie betrachten die schlanken und durchtrainierten Models die Werbung für Kleidung, Sport- und Fitnessprodukte machen.“

Zusatzmaterial 4

Itemkennwerte.

**Item**	***M (SD)***	**Kurtosis**	**Schiefe (SE)**
1	5,30 (2,46)	–1,40	–0,32
2	5,08 (2,05)	–0,93	–0,41
3	4,75 (1,38)	0,64	–0,11
4	4,41 (1,92)	–0,81	0,05
5	4,88 (2,49)	–1,42	–0,06
6	4,65 (2,60)	–1,36	0,16

Abkürzungen: *M*, Mittelwert; *SD*, Standardabweichung.

Zusatzmaterial 5

Respezifikationen.

**Modell**	**MI**	**SB-χ^2**	***df***	**CFI**	**TLI**	**RMSEA [90% *KI*]**	**SRMR**
Original		152,41***	9	0,944	0,906	0,181 [0,157, 0,207]	0,035
Original + Item 5 ~~ Item 6	143,49	63,33***	8	0,980	0,963	0,114 [0,089, 0,141]	0,021
Original + Item 5 ~~ Item 6 + Item 2 ~~ Item 4	37,92	33,79***	7	0,991	0,980	0,083 [0,056, 0,112]	0,017
Original + Item 5 ~~ Item 6 + Item 2 ~~ Item 4 + Item 4 ~~ Item 5	12,94	22,80***	6	0,994	0,986	0,070 [0,041, 0.102]	0,017

Strukturgleichungsmodellierungen mit robuster Maximum-Likelihood Schätzung mit Satorra-Bentler Korrektur.
Abkürzungen: MI, Modifikationsindex; SB-χ^2, Chi-Quadrat-Teststatistik mit Satorra-Bentler Korrektur; CFI, comparative fit index; TLI, Tucker-Lewis index; RMSEA, root mean square error of approximation; KI, Konfidenzintervall; SRMR, standardized root mean square residual.
$^{***}p < 0{,}001$.
